# Camrelizumab plus CAPOX versus CAPOX for HER2-negative gastric or gastro-oesophageal junction adenocarcinoma: a cost-effectiveness analysis

**DOI:** 10.3389/fphar.2026.1854496

**Published:** 2026-07-08

**Authors:** Ling Fang, Longfeng Zhang, Lisheng Huang, Xiumei Lin, Yuling Zhang, Zhiwei Zheng

**Affiliations:** 1 Department of Pharmacy, Cancer Hospital of Shantou University Medical College, Shantou, Guangdong, China; 2 Department of Thoracic Oncology, Clinical Oncology School of Fujian Medical University, Fujian Cancer Hospital, Fuzhou, Fujian, China; 3 Department of Radiation Oncology, Cancer Hospital of Shantou University Medical College, Shantou, China; 4 Department of Outpatient, Clinical Oncology School of Fujian Medical University, Fujian Cancer Hospital, Fuzhou, Fujian, China; 5 Pharmacy Department, Shantou University Medical College, Shantou, Guangdong, China

**Keywords:** advanced gastric cancer, advanced gastro-oesophageal junction adenocarcinoma, camrelizumab, CAPOX, cost-effectiveness analysis

## Abstract

**Objective:**

This study was to assess the cost-effectiveness of camrelizumab in combination with capecitabine plus oxaliplatin (CAPOX) compared to CAPOX for human epidermal growth factor receptor 2 (HER2)-negative advanced gastric or gastro-oesophageal junction adenocarcinoma (GC/GEJC) in China.

**Methods:**

A partitioned survival model was constructed to simulate the 10-year disease progression with HER2-negative advanced GC/GEJC treatment scenarios. Survival data for the model were sourced from NCT03813784 clinical trial. The costs considered in the model included drug costs, management costs for severe adverse events, subsequent treatment costs, and best supportive care costs. Health outcomes were measured in quality-adjusted life year (QALY). The willingness-to-pay (WTP) threshold was defined as 41,876.05 USD per QALY. Sensitivity analyses were conducted to assess the robustness of the model results.

**Results:**

In the overall study population, the camrelizumab plus CAPOX had an incremental cost of 10,688.44 USD and an additional 1.13 QALYs compared to the CAPOX group. This resulted in an incremental cost-effectiveness ratio (ICER) of 9,458.80 USD per QALY. In the PD-L1 positive subgroup, the addition of camrelizumab to CAPOX in this subgroup led to an incremental QALY gain of 1.17, resulting in an ICER of 9,183.07 USD per QALY. The ICER values observed in both groups were found to be below the WTP thresholds. The results of the one-way sensitivity analysis showed that changes in the input parameters did not substantially alter the conclusion. The probabilistic sensitivity analysis indicated that the probability of camrelizumab plus CAPOX being cost-effective was 100% at the WTP threshold of 41,876.05 USD per QALY.

**Conclusion:**

Camrelizumab with CAPOX demonstrates cost-effectiveness as a first-line treatment option for patients with advanced HER2-negative GC/GEJC. This regimen offers promising clinical outcomes while also being economically feasible within the context of China’s healthcare system.

## 1 Introduction

Gastric cancer or gastroesophageal junction adenocarcinoma (GC/GEJC) represents major public health challenges worldwide and rank among the most common and deadly cancers globally (Wang et al., 2024). In 2022, there were 968,780 new cases of gastric cancer reported globally, account for 4.9% of all new cancer diagnoses. The mortality attributed to gastric cancer reached 660,180 deaths, accounting for 6.8% of all cancer-related fatalities worldwide (Bray et al., 2024). China has the highest disease burden of GC/GEJC globally, making it a significant health concern within the East Asia region (Jiang et al., 2024). In 2020, a total of 478,000 new cases of gastric cancer were diagnosed in China, leading to 373,000 associated deaths. This accounted for 43.9% of the global incidence of new gastric cancer cases and 48.6% of global gastric cancer-related mortality (Yin and Zhang, 2025). It is concerning that a large proportion of patients are often diagnosed at an advanced stage, rendering the tumor unresectable, locally advanced, or metastatic. This unfortunate scenario has resulted in a 5-year overall survival rate of less than 10% (Budginaite et al., 2023).

For decades, platinum-based chemotherapy, most commonly the capecitabine plus oxaliplatin (CAPOX) regimen, has served as the first-line treatment for human epidermal growth factor receptor 2 (HER2) negative advanced GC/GEJC (Moehler et al., 2021). Despite significant advancements in chemotherapy, the efficacy of chemotherapy as a monotherapy has reached a therapeutic plateau with limited further improvement (Wang et al., 2023). The median overall survival (OS) rate remains approximately 1 year, while the median progression-free survival (PFS) stands at approximately 6 months (Lordick et al., 2022). Immune checkpoint inhibitors (ICIs), most notably programmed cell death protein 1/programmed cell death ligand 1 (PD-1/PD-L1)-targeted monoclonal antibodies, have fundamentally reshaped the management of advanced GC/GEJC (Chen et al., 2025). The superior clinical efficacy of first-line PD-1 inhibitor-chemotherapy combination over chemotherapy alone in advanced GC/GEJC was first robustly confirmed in the pivotal CheckMate 649 trial (Janjigian et al., 2021). This conclusion has been robustly validated by a series of subsequent pivotal phase 3 clinical trials, including KEYNOTE-859, ORIENT-16 and RATIONALE-305, which collectively establish immune checkpoint inhibitor-based combination therapy as the new first-line standard of care for patients with HER2-negative advanced GC/GEJC (Rha et al., 2023; Xu et al., 2023; Qiu et al., 2024).

A humanized anti-PD-1 monoclonal antibody, camrelizumab is independently developed in China, with its binding epitopes and pharmacokinetic properties rationally optimized to achieve enhanced anti-tumor immune activity (Sun et al., 2024). The recently published final analysis of a phase 3 clinical trial (ClinicalTrials.gov ID: NCT03813784) established that first-line treatment with camrelizumab plus CAPOX chemotherapy demonstrated a statistically significant improvement in median OS, from 12.1 months with CAPOX monotherapy to 14.2 months in the overall intention-to-treat population. In the PD-L1-positive subgroup, this combination regimen delivered an even more pronounced survival benefit, with mOS increasing from 12.5 months in the chemotherapy-alone arm to 15.3 months in the camrelizumab combination arm. These survival improvements were coupled with statistically significant enhancements in key secondary efficacy endpoints, including PFS and objective response rate (ORR), while the combination regimen exhibited a well-tolerated and manageable safety profile with no new or unexpected safety signals (Peng et al., 2026). These findings established camrelizumab plus CAPOX as a preferred first-line regimen for Chinese patients with advanced HER2-negative GC/GEJC.

While the clinical efficacy of camrelizumab plus CAPOX has been validated in randomized controlled trials, its clinical adoption and long-term value depend on both survival benefits and economic affordability. Cost-effective, budget-controlled cancer treatment strategies are critical for alleviating China’s national cancer burden (Abdul-Sater et al., 2021). While several cost-effective studies have evaluated other PD-1 inhibitor plus chemotherapy regimens for advanced GC/GEJC in China, to date, no cost-effective study specifically assessing camrelizumab plus CAPOX for this indication has been published. This critical evidence gap directly hinders evidence-based clinical decision-making and medical insurance policy optimization for this commonly prescribed regimen.

Furthermore, some existing cost-effective studies for advanced GC/GEJC rely on interim OS data with median follow-up of 12–24 months, which cannot fully capture the long-term survival plateau characteristic of ICIs. This systematically underestimates the health benefits of immunotherapy and may overestimate incremental cost-effectiveness ratios (ICERs). Additionally, most studies used the drug prices from 2022 or earlier that do not reflect the significant price reductions achieved through recent national centralized procurement negotiations.

Therefore, from the perspective of the Chinese healthcare system, we constructed a partitioned survival model to conduct the first pharmacoeconomic evaluation of camrelizumab plus CAPOX versus CAPOX alone. Our study addresses all the aforementioned limitations by using the final OS data from NCT03813784 (median follow-up 36.2 months) and incorporating the latest 2026 national centralized procurement drug prices. The findings will provide the most up-to-date and clinically relevant evidence to support clinical practice guidelines and medical insurance reimbursement decisions.

## 2 Methods

### 2.1 Study model design

**FIGURE 1:**
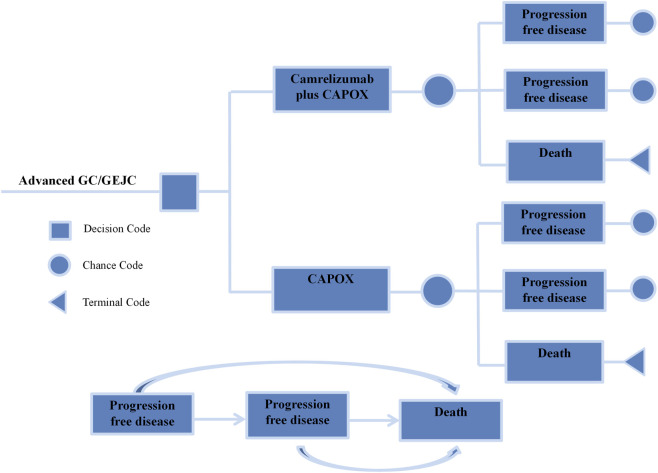
Structure of the three-state partitioned survival model. The model consists of three distinct health states that are mutually exclusive. At each decision node, various options are depicted, resulting in diverse outcomes at chance nodes based on probabilities (CAPOX:capecitabine 1,000 mg/m^2^ orally twice daily on days 1–14 plus oxaliplatin 130 mg/m^2^ intravenously on day 1, every 3 weeks).

A partitioned survival model (PSM) was developed to simulate the natural disease course over a 10-year period for patients with advanced HER2-negative GC/GEJC. The model framework is structured with three mutually exclusive, non-overlapping health states that collectively cover the full disease trajectory of advanced HER2-negative GC/GEJC: progression-free disease, progressive disease (PD), and death. Progression-free disease was defined as the duration from the first-line of therapeutic intervention to either the progression of the underlying disease or the occurrence of death in the patient. Disease progression was typically characterized by a substantial increase of at least 20% in the aggregate dimensions of identified target lesions or the appearance of new lesions. Patients entering this health state remained alive with confirmed disease progression following first-line treatment, and received second-line palliative anti-tumor therapy in conjunction with standard best supportive care, in alignment with clinical practice guidelines for advanced gastric cancer in China. For the survival analyses incorporated in the model, death was specified as the definitive endpoint to quantify the time from treatment commencement to patient death. The entire pharmacoeconomic model was constructed using TreeAge Pro 2022 software, with the model structure systematically depicting all relevant decision nodes, chance nodes, and terminal nodes corresponding to the clinical decision-making and disease progression pathways. At each decision node, different options are presented, leading to various outcomes at chance nodes based on probabilities. A half-cycle correction was performed for all state transitions and survival estimations in the mode. PSM calculates the proportion of patients in each health state at each cycle directly from the area under the OS and PFS curves derived from the NCT03813784 trial. The proportion of patients in the PFS state was estimated from the PFS curve, the proportion of deceased patients was estimated from the OS curve, and the proportion of patients in the PD state was calculated as the difference between the OS and PFS proportions. Figure 1 illustrates the model structure used in this study. For patients in the progression-free survival state, routine follow-up was scheduled every 6 weeks, including physical examination, complete blood count, liver and renal function tests, and contrast-enhanced computed tomography (CT) of the abdomen and pelvis. Upon entering the progressive disease state, patients initiated second-line palliative chemotherapy within 2 weeks of radiologically confirmed disease progression. The base-case model assumed paclitaxel (175 mg/m^2^ intravenously every 3 weeks) as the standard second-line regimen. Patients who progressed after second-line treatment received best supportive care only. Death was defined as the terminal health state, and a one-time terminal care cost was included to account for end-of-life medical expenses.

In compliance with international pharmacoeconomic research standards from the Chinese Guidelines for Pharmacoeconomic Evaluations (2020 edition) (Liu et al., 2020), key structural parameters of the model were predefined as follows:First, the time horizon was established at 10 years, a timeframe sufficient to cover the entire lifetime survival trajectory of patients with advanced GC/GEJC, as published data confirm the 5-year overall survival rate of this population is less than 10%. Second, the model cycle length was specified as 21 days, consistent with the clinical administration cycle of the CAPOX backbone chemotherapy regimen. All direct medical costs and health utility outcomes were discounted at an annual rate of 5%.

All costs included in this study were standardized to United States dollars (USD) using the 2025 annual average exchange rate published by the Bank of China, with a conversion specification of 1 USD = 7.14 Chinese Yuan (RMB). The willingness-to-pay (WTP) threshold for cost-effectiveness evaluation was established as 3 times China’s 2025 *per capita* gross domestic product (GDP), corresponding to 41,876.05 USD per quality-adjusted life year (QALY)) (National Bureau of statistic, 2026).

### 2.2 Target population and treatment

The target population of this study was consistent with the inclusion criteria of the NCT03813784 trial: adult patients aged ≥18 years, with histologically or cytologically confirmed unresectable locally advanced, recurrent, or metastatic GC/GEJC, Eastern Cooperative Oncology Group (ECOG) performance status of 0 or 1, no prior systemic chemotherapy for advanced disease, and HER2-negative status (Peng et al., 2026).

The study compared two treatment strategies: the combination of camrelizumab with CAPOX (camrelizumab plus CAPOX group) versus CAPOX alone (CAPOX group). Patients in the camrelizumab plus CAPOX group received a regimen consisting of camrelizumab (200 mg intravenously every 3 weeks) in combination with CAPOX (capecitabine 1,000 mg/m^2^ orally twice daily on days 1–14 of each 21-day treatment cycle, and oxaliplatin 130 mg/m^2^ intravenously on day 1 of each cycle) for a total of four to six cycles, followed by camrelizumab monotherapy. Patients in the CAPOX group received capecitabine and oxaliplatin alone (Peng et al., 2026). The median exposure durations in the camrelizumab plus CAPOX group were 5.8 months (interquartile range [IQR] 3.5–10.4 months) for camrelizumab, 3.9 months (IQR 2.7–4.0 months) for capecitabine, and 4.1 months (IQR 2.9–4.3 months) for oxaliplatin (Peng et al., 2026). In comparison, the median treatment durations in the CAPOX group were 3.9 months (IQR 2.3–4.8 months) for capecitabine and 4.1 months (IQR 2.5–4.9 months) for oxaliplatin (Peng et al., 2026).

After discontinuation of the primary study treatment, 46.3% (82 out of 177) of patients in the camrelizumab plus CAPOX group and 59.9% (209 out of 349) of patients in the CAPOX group received at least one subsequent antitumor therapy (Peng et al., 2026). According to the NCT03813784 clinical trial, over 50% of the patients in the study received systemic chemotherapy as a subsequent treatment (Peng et al., 2026). As part of the cost analysis, our study made certain assumptions regarding the subsequent treatment regimens. It was assumed that the second-line therapy after progression was the paclitaxel regimens (175 mg/m^2^ every 3 weeks), in accordance with the Chinese Society of Clinical Oncology guidelines (CSCO)and the NCCN Clinical Practice Guidelines In Oncology (Ajani et al., 2025). Furthermore, following failure of second-line regimens, our study assumed that best supportive care would be the most appropriate regimen in this scenario.

Treatment-related grade 3 to 4 serious adverse events (SAEs) with an incidence rate exceeding 5%in the phase III NCT03813784 trial, were predefined and incorporated into the analytical model. Grade 1-2 adverse events were excluded from the model for several reasons. Firstly, grade 1-2 adverse events have minimal impact on the health-related quality of life of patients, and the corresponding disutility value is negligible. Secondly, grade 1-2 adverse events typically do not necessitate additional medical intervention, resulting in minimal additional medical costs.

### 2.3 Survival data analysis and extrapolation

Individual patient data (IPD) corresponding to PFS and OS were digitally retrieved from the published Kaplan-Meier (K-M) curves of trial NCT03813784, with full digitization performed via GetData Graph Digitizer 2.26 software and a total of 140 standardized sampling points captured for each K-M survival curve (Hoyle and Henley, 2011). Parameter distribution fitting was conducted on the extracted survival data to determine the most appropriate statistical distribution. We evaluated a panel of candidate parametric survival distributions for model extrapolation, including Exponential, Weibull, Gamma, Gompertz, Log-normal, and Log-logistic models. The optimal fitting distribution for each survival endpoint was ultimately selected based on standardized goodness-of-fit criteria, with the lowest Akaike Information Criterion (AIC) and Bayesian Information Criterion (BIC) values defined as the primary selection criterion (Cai et al., 2024). Quantitatively, the log-logistic distribution consistently yielded the lowest AIC and BIC values across all four survival endpoints (OS and PFS for both treatment arms), outperforming all other candidate models. Complete AIC and BIC values for all tested distributions are provided in Supplementary Tables S2, S3. In addition to evaluating the goodness of fit using statistical criteria such as AIC and BIC, we further validated the fitted model through visual inspection of the survival curves. Visually, the log-logistic curve most accurately reproduced the observed survival trajectory throughout the entire follow-up period, particularly capturing the late-stage survival plateau characteristic of immune checkpoint inhibitor therapy, which other distributions tended to underestimate. Full fitted survival curves for all candidate distributions are presented in Supplementary Figures S1, S2.

Within the in-trial follow-up period of the NCT03813784 trial, survival probabilities were directly retrieved from the trial’s published K-M survival curves, which were further used to calculate the proportion of patients allocated to each mutually exclusive health state in our partitioned survival model. For the long-term extrapolation period beyond the trial’s follow-up, we applied the optimally fitted log-logistic parametric distribution to extrapolate survival outcomes and construct the corresponding survival function, denoted as S(t). The survival function of the log-logistic distribution at time t is defined as: S(t) = 1/(1 + λt^γ^) (Ishak et al., 2013). The final fitted shape (γ) and scale (λ) parameters are reported in Table 1. The log-normal distribution as the next best fitting function was also used to project lifetime survival curves in our model. The detailed results of this model were presented in Supplementary Table S4.

**TABLE 1:** Survival parameters input.

Variable	Overall population		PD-L1 positive population	
	Camrelizumab plus CAPOX group	CAPOX group	Camrelizumab plus CAPOX group	CAPOX group
OS- shape(γ)	1.869	1.960	1.684	1.895
OS-scale(λ)	0.00666	0.00789	0.00953	0.00816
PFS-shape(γ)	1.733	2.186	1.654	2.173
PFS-scale(λ)	0.0350	0.0253	0.0325	0.0219

### 2.4 Costs and health utility input

The analysis considered only direct medical costs from the perspective specified for the study. The cost components included in this study were categorized into four core areas: drug costs, disease management costs, costs related to the management of SAEs, and costs associated with subsequent anti-tumor treatment and best supportive care. The drug pricing data presented here is sourced from the Yaozh database, which provides information on the latest median bidding prices in 2026 across different provinces and cities in China. Dosages of drugs were calculated based on the average body surface area of 1.72 m^2^ for Chinese adult patients. For disease management costs, we accounted for expenses related to routine laboratory assessments, including complete blood count, liver and renal function tests, and tumor marker detection, as well as necessary computed tomography imaging. Regarding the management costs of SAEs, the included SAEs were platelet count decreased, neutrophil count decreased, white blood cells (WBC) decreased and anaemia. The incidence of each eligible SAEs was directly extracted from the safety data set of the NCT03813784 trial. The one-time treatment cost for each episode of SAEs was obtained from the literature that aligned with the research setting of the study. Finally, costs for subsequent second-line treatment, best supportive care and Follow-up cost were also incorporated into the analytical model. The proportions of patients receiving subsequent therapy (46.3% for the experimental arm and 59.9% for the control arm) were taken directly from the NCT03813784 trial follow up data. Subsequent treatment cost was set as a fixed per patient value weighted by these trial based proportions, using paclitaxel 175 mg/m^2^ every 3 weeks with dosage calculated from the average body surface area of Chinese patients. The number of treatment cycles was determined by state transition probabilities in the partitioned survival model.

Quality-adjusted life years (QALYs) were utilized as the primary measure of health outcome in this study. For each model cycle, QALYs were computed as the product of the length of time patients remained in each predefined health state and the corresponding health utility weight for that state. Health utility values for the PFS and PD health states were sourced from the study by Tsuchiya et al. (2002), which developed the first standardized EQ-5D population value set for East Asian populations using the internationally recognized time trade-off method. While this study was conducted in a Japanese population, health preference structures have been shown to be highly similar between Chinese and Japanese populations for cancer-related health states, and these values have been widely adopted in pharmacoeconomic evaluations of advanced gastric cancer in China. Importantly, the utility values were specifically assessed in patients with advanced gastric cancer receiving first-line systemic therapy, which exactly matches the disease stage and treatment context of our study population. Specifically, a utility value of 0.80 (95% confidence interval [CI]: 0.63–0.95) was applied to the PFS state, and a utility value of 0.58 (95% CI: 0.46–0.69) was assigned to the PD state. The utility value for the death state was set at 0, reflecting the absence of quality of life in this state. The robustness of these utility assumptions was verified through extensive sensitivity analyses where both values were varied across their full 95% confidence intervals. The cost and utility values used in this study are presented in Table 2.

**TABLE 2:** The input parameters.

Parameters	Baseline	Range		Distribution	Source
	Value	Minimum	Maximum		
Clinical inputs					
SAEs rate of camrelizumab plus CAPOX group [No. (%)]					
Platelet count decreased	17 (9.6)	-	-	Beta	Peng et al. (2026)
Neutrophil count decreased	29 (16.4)	-	-	Beta	Peng et al. (2026)
WBC decreased	9 (5.1)	-	-	Beta	Peng et al. (2026)
Anaemia	16 (9.0)	-	-	Beta	Peng et al. (2026)
SAEs rate of CAPOX group [No. (%)]					
Platelet count decreased	61 (17.5)	-	-	Beta	Peng et al. (2026)
Neutrophil count decreased	58 (16.6)	-	-	Beta	Peng et al. (2026)
WBC decreased	21 (6.0)	-	-	Beta	Peng et al. (2026)
Anaemia	26 (7.4)	-	-	Beta	Peng et al. (2026)
Proportion of subsequent systemic chemotherapy treatment					
Camrelizumab plus CAPOX group	0.463	-	-	Beta	Peng et al. (2026)
CAPOX group	0.599	-	-	Beta	Peng et al. (2026)
Costs (USD)					
Camrelizumab (200 mg)	360.87	288.70	433.04	Gamma	YaoZH (2026)
Capecitabine (500 mg)	0.39	0.31	0.47	Gamma	YaoZH (2026)
Oxaliplatin (50 mg)	6.55	5.24	7.86	Gamma	YaoZH (2026)
Paclitaxel (30 mg)	5.68	4.54	6.82	Gamma	YaoZH (2026)
Platelet count decreased	1,045.20	836.16	1,254.24	Gamma	Yan et al. (2026)
Neutrophil count decreased	539.54	431.63	647.45	Gamma	Yan et al. (2026)
WBC decreased	211.06	168.85	253.27	Gamma	Zhou et al. (2025)
Anaemia	336.97	269.58	404.36	Gamma	Zhou et al. (2025)
Best supportive care	248.00	198.40	297.60	Gamma	Lin et al. (2025)
Follow-up cost per cycle	55.60	44.48	66.72	Gamma	Lin et al. (2025)
Laboratory examinations per cycle	92.50	74.00	111.00	Gamma	Zhang et al. (2025)
Computer tomography per cycle	105.90	84.72	127.08	Gamma	Zhang et al. (2025)
Drug administration per unit	37.31	29.85	44.77	Gamma	Wang et al. (2026)
Terminal care cost in end-of-life	1,492.49	1,193.99	1790.99	Gamma	Zou et al. (2025)
Utility					
Progression-free disease	0.80	0.64	0.96	Beta	Tsuchiya et al. (2002)
Progressive disease	0.58	0.46	0.70	Beta	Tsuchiya et al. (2002)
Platelet count decreased	0.09	0.07	0.11	Beta	Lai et al. (2024)
Neutrophil count decreased	0.15	0.12	0.18	Beta	Lai et al. (2024)
WBC decreased	0.20	0.16	0.24	Beta	Lai et al. (2024)
Anaemia	0.073	0.06	0.09	Beta	Lai et al. (2024)
Body surface area (m^2^)	1.72	1.38	2.06	Beta	Huang et al. (2023)
Discount rate	5%	0	8%	Beta	Liu et al. (2020)

### 2.5 Sensitivity analysis

To comprehensively validate the robustness of our base-case analysis findings and quantify the impact of parameter uncertainty on the final outputs of the established pharmacoeconomic model, we performed a series of systematic sensitivity analyses. For the one-way deterministic sensitivity analysis, each core input parameter was varied independently within its predefined clinically and economically plausible ranges: a ±20% fluctuation was set for all cost-related parameters, with an identical ±20% variation applied to health utility values, incidence rates of treatment-related adverse events, and the annual discount rate. This analysis was designed to delineate the independent influence of individual parameter changes on the ICER, with results visualized via a tornado diagram.

Concurrently, a probabilistic sensitivity analysis (PSA) with 1000-iteration second-order Monte Carlo simulation was conducted to account for the joint uncertainty of all model input parameters, where appropriate probability distributions were assigned to each category of model parameters in accordance with internationally recognized pharmacoeconomic research norms: Gamma distributions were adopted for all cost parameters given their non-negative and right-skewed numerical characteristics, while Beta distributions, which are inherently suitable for variables constrained between 0 and 1, were applied to health utility values, adverse event incidence rates, and the discount rate, with the outputs of the PSA ultimately presented as a cost-effectiveness scatter plot and a cost-effectiveness acceptability curve.

## 3 Results

### 3.1 Base-case analysis

Base-case analysis results are summarized in Table 3. In the overall study population, first-line camrelizumab combined with CAPOX chemotherapy was associated with an incremental total cost of 10,688.44 USD, and yielded an QALY gain of 1.13, relative to CAPOX monotherapy. This corresponded to an ICER of 9,458.80 USD per QALY gained.

**TABLE 3:** The base case results.

Variable	Overall population		PD-L1 positive population	
	Camrelizumab plus CAPOX group	CAPOX group	Camrelizumab plus CAPOX group	CAPOX group
Cost (USD)	32,671.69	21,983.25	31,913.69	21,169.50
Incremental cost (USD)	10,688.44	—	10,744.19	—
QALYs	3.20	2.07	3.17	2.00
Incremental QALY	1.13	—	1.17	—
ICER (USD/QALY)	9,458.80	—	9,183.07	—

In the PD-L1-positive subgroup, the camrelizumab plus CAPOX regimen incurred a total cost of 31,913.69 USD, representing an incremental cost of 10,744.19 USD compared with the CAPOX alone group. Meanwhile, the combination regimen generated an incremental QALY gain of 1.17 (3.17 QALYs vs. 2.00 QALYs for CAPOX), resulting in an ICER of 9,183.07 USD per QALY gained.

Notably, the ICER values of the camrelizumab plus CAPOX regimen in both the overall population and PD-L1-positive subgroup were markedly lower than the WTP threshold. Both overall and PD-L1-positive subgroup ICERs were well below the two times *per capita* GDP threshold (27,917.37 USD/QALY) and the three times *per capita* GDP WTP threshold (41,876.05USD/QALY). These findings confirm that the addition of camrelizumab to standard CAPOX chemotherapy is a cost-effective first-line therapeutic strategy for patients with HER2-negative advanced GC/GEJC, with even more favorable economic performance observed in the PD-L1-positive population.

### 3.2 The one-way sensitivity analysis results

**FIGURE 2:**
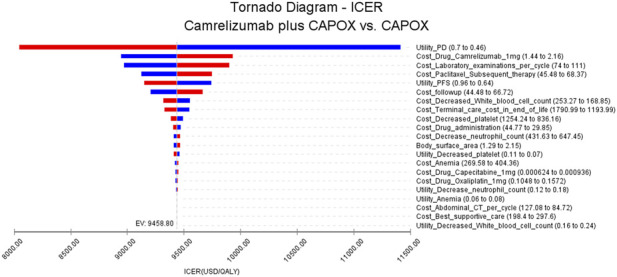
The results of the one-way sensitivity analysis for the overall population.

**FIGURE 3:**
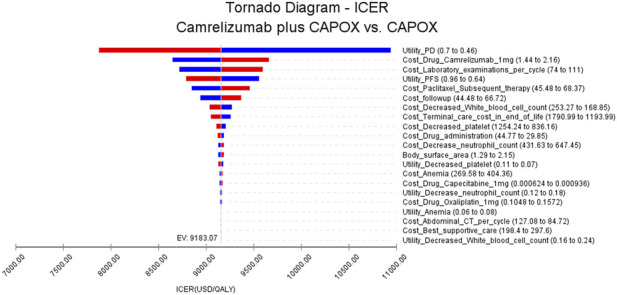
The results of the one-way sensitivity analysis for the PD-L1 positive subgroup.

One-way sensitivity analysis was conducted to verify the robustness of base-case results and evaluate the influence of individual parameter uncertainty on the ICER of camrelizumab plus CAPOX versus CAPOX. The results for the overall population and PD-L1 positive subgroup displayed in Figures 2, 3, respectively. In both cohorts, the health utility of progressive disease state imposed the greatest impact on ICER, followed by the cost of camrelizumab, the laboratory examination cost, the cost of paclitaxel subsequent treatment, and progression-free survival health utility, which were the sensitive parameters that affect the results of the model. Meanwhile, parameters related to the costs of follow-up, adverse events, chemotherapy drug costs, body surface area, and terminal care cost in end-of-life showed only limited effects on ICER, with consistent trends observed across the two populations.

Critically, the ICER values for the camrelizumab plus CAPOX combination regimen remained below the WTP threshold across the entire fluctuation range of all model input parameters, confirming that our base-case cost-effectiveness conclusion was robust and not significantly affected by the uncertainty of individual model parameters.

### 3.3 The probabilistic sensitivity analysis results

**FIGURE 4:**
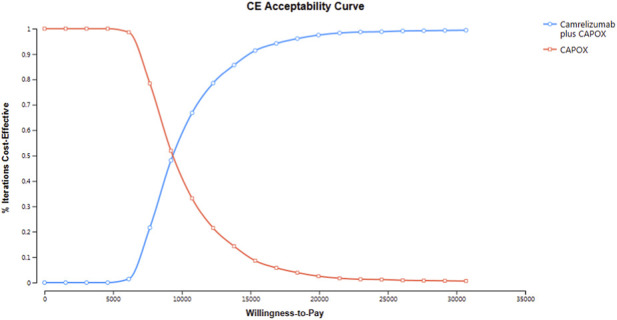
The cost-effectiveness acceptability curve result for the overall population.

**FIGURE 5:**
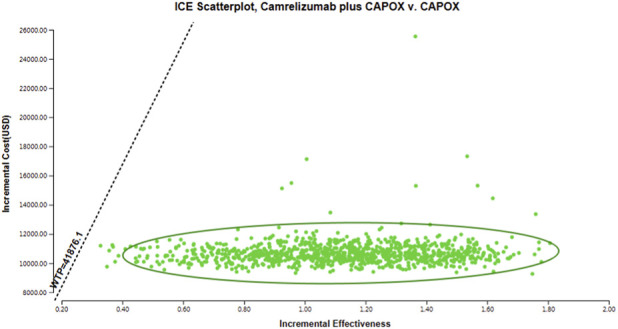
The scatter plot result for the overall population.

The cost-effectiveness acceptability curves (Figure 4 for overall population; Supplementary Figure S3 for PD-L1 positive subgroup) showed a consistent upward trend in the cost-effectiveness probability of the combination regimen with increasing WTP thresholds. The camrelizumab plus CAPOX had a 100% cost-effectiveness probabilit both in the overall population and the PD-L1 positive subgroup at the 41,876.05 USD per QALY WTP threshold. Notably, even at the more stringent two times GDP *per capita* WTP threshold, the cost-effectiveness probability remained extremely high at 99.1% in the overall population and 99.6% in the PD-L1-positive subgroup. The cost-effectiveness scatter plots (Figure 5; Supplementary Figure S4) further confirmed that most iterative sampling points were located in the first quadrant and fell below the WTP threshold, indicating the regimen yielded incremental health benefits at an acceptable cost in nearly all simulated scenarios. Collectively, the PSA findings robustly validated that our base-case cost-effectiveness conclusion was stable against the joint uncertainty of all model parameters, and camrelizumab plus CAPOX maintained a high likelihood of being cost-effective for both study cohorts within clinically reasonable WTP thresholds.

## 4 Discussion

Advanced GC/GEJC represents a significant disease burden in China, necessitating first-line treatment strategies that optimize survival outcomes, clinical feasibility, and cost-effectiveness (Hairoula et al., 2025). ICIs have fundamentally revolutionized the first-line treatment paradigm for HER2-negative advanced GC/GEJC. Among these regimens, camrelizumab combined with CAPOX chemotherapy has established itself as a highly promising therapeutic strategy, supported by robust efficacy and safety data from the pivotal phase III clinical trial (ClinicalTrials.gov identifier: NCT03813784). However, comprehensive pharmacoeconomic evaluations specific to the Chinese context are scarce. Therefore, there is a need cost-effectiveness studies to inform decision-making and optimize treatment outcomes for advanced GC/GEJC in China.

In this study, we adopted a partitioned survival model to evaluate the long-term cost-effectiveness of camrelizumab combined with CAPOX chemotherapy versus CAPOX for HER2-negative advanced GC/GEJC, with stratified analyses performed for both the overall intention-to-treat study cohort and the PD-L1-positive patient subgroup. Our base-case analysis revealed that adding camrelizumab to CAPOX yielded incremental QALY gains of 1.13 in the overall population and 1.17 in the PD-L1-positive subgroup, with corresponding ICERs calculated as 9,458.80 USD per QALY and 9,183.07 USD per QALY, respectively. Collectively, these findings demonstrate that incorporating camrelizumab into first-line CAPOX regimens delivers significant clinical survival benefits with a controllable incremental cost, positioning it as a cost-effective and favorable first-line therapeutic option for Chinese patients with advanced GC/GEJC with the perspective of the Chinese healthcare system.

Our base-case findings were validated through sensitivity analysis to assess the robustness of our results. Tornado diagrams generated from the one-way sensitivity analysis highlighted the health utility value of the PD state as the most influential parameter driving variability in the ICER for both cohorts, followed by the cost of camrelizumab, the laboratory examination cost, and the cost of paclitaxel subsequent treatment. However, all ICER values for the combination regimen remained below the WTP threshold, even when considering the full range of fluctuation in all input parameters. The significant influence of disease progression health state utility in patients with advanced gastric cancer is both biologically and clinically justified. Despite disease progression, individuals often survive for an extended period, emphasizing the importance of considering health-related quality of life during disease (Sowell et al., 2025). This impact is particularly notable as it directly contributes to the accumulation of total quality-adjusted life years over the specified model time horizon. The impact parameters included in the analysis were the cost of subsequent therapy and the cost of camrelizumab. The sensitivity of camrelizumab pricing is consistent with existing literature on the importance of drug acquisition cost in the cost-effectiveness of ICIs therapies (Xiang et al., 2024). This underscores the significance of China’s national medical insurance negotiation policies, which have effectively reduced the clinical cost of PD-1 inhibitors, in facilitating the cost-effective utilization of these regimens in clinical practice (Cao et al., 2024). Furthermore, the sensitivity of subsequent treatment costs reflects the variability in progression therapeutic strategies in real-world settings. This suggests that the implementation of standardized, guideline-concordant second-line and later treatments can enhance the long-term economic value of first-line camrelizumab plus CAPOX therapy. In contrast, parameters including the management costs and health utility disutilities of grade ≥3 SAEs, the costs of CAPOX chemotherapy, body surface area, and cost of follow-up and imaging cost exerted negligible effects on model outputs.

Cost-effectiveness acceptability curves derived from the PSA consistently demonstrated that the probability of camrelizumab plus CAPOX being deemed cost-effective rose in a stepwise manner with increasing WTP thresholds. In parallel, the incremental cost-effectiveness scatter plots from the 1,000 Monte Carlo simulation iterations confirmed that the vast majority of simulated data points fell below the WTP threshold for the Chinese healthcare setting. The PD-L1-positive patient subgroup consistently exhibited a higher probability of cost-effectiveness across all evaluated WTP thresholds, which aligns directly with the more favorable base-case ICER observed in this biomarker-enriched population. These findings further validate the economic value of the camrelizumab plus CAPOX combination regimen, particularly in PD-L1-positive patients, and emphasize the critical role of biomarker-guided treatment strategies in optimizing the cost-effective allocation of healthcare resources within oncology practice. In contrast to the indiscriminate use of ICI therapy in all patients with advanced GC/GEJC, the implementation of PD-L1 guided treatment represents a more targeted approach that directs costly ICI therapies to those individuals most likely to benefit clinically and economically (Huo et al., 2023). This personalized treatment strategy mitigates unnecessary expenditures on patients unlikely to respond to therapy, thus optimizing resource allocation and cost-effectiveness (Stelzer et al., 2020). By prioritizing PD-L1 positive patients for camrelizumab plus CAPOX therapy, a greater total of QALYs can be achieved compared to the unselected administration of ICIs therapy across all patients. This approach aligns with the fundamental objective of efficient healthcare resource utilization and underscores the importance of evidence-based strategies in guiding treatment decisions (Velikova et al., 2024).

In this study, we utilized the WTP threshold recommended by the Chinese Pharmacoeconomic Evaluation Guidelines (2020 edition) (Liu et al., 2020), which is set at 3 times China’s projected 2025 *per capita* gross domestic product (GDP), to assess the economic value of the camrelizumab plus CAPOX combination regimen across various decision-making scenarios. At the WTP threshold of 3 times national *per capita* GDP, a near 100% probability of cost-effectiveness for the camrelizumab plus CAPOX regimen was demonstrated by our analysis across both the overall study population and the PD-L1-positive tumor patient subgroup. While the three times *per capita* GDP threshold remains the officially WTP for pharmacoeconomic evaluations in China per the 2020 national guidelines, the two times the GDP *per capita* as the WTP threshold more accurately reflects the shifting paradigm of healthcare resource allocation in China, particularly amid ongoing efforts to optimize national medical insurance budgets and enhance the affordability of innovative anticancer therapies. Our base-case analysis revealed that the ICERs for camrelizumab plus CAPOX were 9,458.80 USD per QALY in the overall population and 9,183.07 USD per QALY in the PD-L1-positive subgroup, values that fell substantially below both the conventional three times threshold and the more conservative two times benchmark. This finding carries profound policy implications, as it confirms that the combination regimen maintains robust cost-effectiveness even when evaluated against more stringent economic standards. Complementing these base-case results, our probabilistic sensitivity analysis demonstrated that the probability of the regimen being cost-effective remained exceptionally high at 99.1% in the overall population and 99.6% in the PD-L1-positive subgroup at the two times GDP *per capita* as the WTP threshold. When considering a WTP threshold set at 1.5 times *per capita* GDP (Ye et al., 2022), the combination regimen maintained a high cost-effectiveness probability of over 90% in the overall population and more than 95% in the PD-L1 positive subgroup. Furthermore, even at the most stringent WTP threshold of 1 time *per capita* GDP (Iino et al., 2022), the ICER of camrelizumab plus CAPOX remained below the threshold limit. The cost-effectiveness probabilities exceeded 85% in the overall population and over 90% in the PD-L1 positive subgroup at this threshold. Collectively, the consistently high cost-effectiveness probability of the regimen across all three tiered thresholds demonstrates its universal applicability across diverse decision-making contexts, from global health policy formulation and national insurance reimbursement review to bedside clinical practice.

The cost-effectiveness of cancer immunotherapies is highly dependent on drug pricing and healthcare system policies, and China’s national medical insurance negotiation mechanism has played a transformative role in improving patient access to innovative anti-tumor drugs. Camrelizumab was first included in the National Reimbursement Drug List (NRDL) in 2020, and has undergone multiple rounds of price negotiations since then, resulting in a cumulative price reduction of over 85% compared to its initial launch price. The 2026 national centralized procurement price used in our base-case analysis reflects the current actual clinical acquisition cost in most Chinese hospitals. To quantify the impact of these price reductions on cost-effectiveness, we conducted a scenario analysis using the pre-negotiation price of camrelizumab ($3,080 per 200 mg). The results showed that the ICER would increase to $112,645.37 per QALY, which far exceeds the WTP threshold of $41,876.05 per QALY. This stark contrast clearly demonstrates that without China’s innovative drug price negotiation policy, camrelizumab plus CAPOX would not be a cost-effective first-line treatment option for advanced GC/GEJC in China.

Regarding PD-L1 testing, while our stratified analysis demonstrated that camrelizumab plus CAPOX has the most favorable economic performance in the PD-L1-positive subgroup, the practical feasibility of routine PD-L1 testing remains a significant challenge in primary medical institutions in China. Currently, PD-L1 testing penetration is estimated to be only 40%–50% in county-level hospitals, primarily due to limited technical capacity, lack of standardized testing protocols, and insufficient pathologists. However, it is important to note that the 2025 CSCO Gastric Cancer Guidelines do not require mandatory PD-L1 testing prior to first-line camrelizumab treatment, as the regimen has demonstrated statistically significant survival benefits in the overall intention-to-treat population. Our economic analysis further supports this guideline recommendation, showing that even without PD-L1 stratification, camrelizumab plus CAPOX remains highly cost-effective in the overall population.

In real-world clinical practice, this means that primary care physicians can safely initiate camrelizumab plus CAPOX treatment without waiting for PD-L1 test results, which avoids treatment delays and improves patient access to effective therapy. For institutions with PD-L1 testing capacity, biomarker-guided treatment can further optimize resource allocation by prioritizing patients who are most likely to derive the greatest clinical and economic benefit. Recent policy initiatives, including the inclusion of PD-L1 testing in the NRDL and the implementation of regional pathology center construction programs, are expected to significantly improve PD-L1 testing accessibility in primary care over the next few years. This will further enhance the value of biomarker-guided treatment strategies and promote more precise and cost-effective cancer care in China.

There is a lack of published studies that have specifically evaluated the cost-effectiveness of camrelizumab in combination with CAPOX for the treatment of advanced GC/GEJC. However, several studies have conducted cost-effectiveness analyses on other PD-1 inhibitors in the management of advanced GC/GEJC. A cost-effectiveness evaluation conducted by Xu et al. investigated the use of tislelizumab plus chemotherapy for advanced GC/GEJC. The study found that the ICER for this treatment option was 33,876.38 USD per QALY gained in China, suggesting that at the WTP threshold of three times the *per capita* GDP of China in 2024, the use of tislelizumab plus chemotherapy as a first-line therapy can be deemed a cost-effective intervention for advanced GC/GEJC (Xu et al., 2024). The ICER of 33,876.38 USD/QALY for tislelizumab plus chemotherapy, which is 3.6-fold higher than our result (9,458.80 USD/QALY). This discrepancy is primarily driven by drug pricing: our study uses the 2026 national centralized procurement price of camrelizumab, which is 32% lower than the 2024 negotiated price of tislelizumab used in their study, outweighing the modest difference in median OS benefit. Another study conducted by Lang et al. examined the cost-effectiveness of cadonilimab in combination with chemotherapy for patients with advanced GC/GEJC in both the USA and China. The study found that the incremental cost-effectiveness ratio (ICER) was 50,582.10 USD per QALY gained in China and 290,498.45 USD per QALY gained in the USA (Lang et al., 2025). These results suggest that utilizing cadonilimab as a first-line therapy may not be considered a cost-effective option at a WTP threshold in both the USA and China (Lang et al., 2025). As a newer PD-1/CTLA-4 bispecific antibody, cadonilimab has not yet undergone multiple rounds of national medical insurance negotiations, resulting in a per-cycle cost 4.2 times higher than camrelizumab. Its numerically greater OS benefit cannot offset this substantial cost disadvantage. Our study utilizes the final OS data with a median follow-up of 36.2 months—the longest among all published cost-effectiveness evaluations of first-line GC/GEJC immunotherapy, allowing more accurate capture of the long-term survival plateau characteristic of ICIs. Additionally, we incorporated the latest 2026 centralized procurement prices, which reflect current real-world clinical costs. Furthermore, to provide a comprehensive context for the relative economic value of different first-line therapeutic strategies for HER2-negative advanced GC/GEJC, we compared our findings with non-PD-1 inhibitor cost-effectiveness analysis of ramucirumab plus paclitaxel as first-line switch maintenance therapy. The ramucirumab plus paclitaxel switch maintenance yielded an incremental QALY gain of 0.15 with an incremental cost of $56,738.52, resulting in an ICER of $373,219.84/QALY in the overall population and $266,259.94/QALY in the PD-L1 CPS ≥5 subgroup, both far exceeding the WTP threshold. By comparison, camrelizumab plus CAPOX yielded an ICER of $9,458.80/QALY, well below the WTP threshold.

Several limitations of this study should be noted. First, uncertainty associated with long-term survival extrapolation must be noted. Although we adopted the optimally fitted log-logistic parametric distribution for survival curve extrapolation, the relatively short follow-up period of the pivotal NCT03813784 trial inevitably introduces inherent uncertainty in the extrapolated long-term survival outcomes of patients in real-world clinical settings. Future studies incorporating piece wise parametric models that integrate trial-derived survival data with longitudinal real world evidence, or prospectively validating extrapolated outcomes through large scale real world database analyses. Secondly, the observed heterogeneity in post-progression therapeutic strategies in clinical practice may introduce potential discrepancies relative to our model assumptions. The base-case analysis presumes paclitaxel monotherapy as the reference second-line regimen. To evaluate this presumption’s influence, we performed scenario analyses examining the irinotecan monotherapy or docetaxel therapy for patients experiencing disease progression. These analyses demonstrated ICER variations spanning $9,323.47-$9,656.22 per QALY across all scenarios, consistently remaining substantially below the predefined willingness-to-pay threshold, thereby affirming the robustness of our primary findings. Thirdly, only grade 3–4 treatment-related SAEs were incorporated into our model. Grade 1–2 adverse events were excluded *a priori*, as these events have been shown to exert minimal to no impact on patients, with negligible associated utility values. Moreover, sensitivity analyses confirmed that the management costs and health utility of grade ≥3 SAEs had no meaningful influence on the final model outputs. Fourth, the health utility values used in our model were derived from a Japanese EQ-5D population value set rather than a Chinese-specific value set. While health preference structures are highly similar between East Asian populations and these values have been widely validated in Chinese gastric cancer cost-effectiveness research, the use of a non-native value set may introduce minor measurement bias. The development and validation of a Chinese-specific EQ-5D value set for advanced cancer patients would improve the accuracy of future cost-effectiveness evaluations. Nevertheless, our extensive sensitivity analyses confirmed that variations in health utility values across their full plausible ranges did not alter the core cost-effectiveness conclusion of our study. Lastly, PD-L1 testing costs were not included in our base-case model. In China, PD-L1 detection is not required prior to camrelizumab based first-line therapy for HER2-negative advanced gastric cancer, and trial-wide survival benefits support treatment use independent of PD-L1 status. However, optional PD-L1 testing in routine clinical practice may incur extra diagnostic costs. To quantify the potential impact of this exclusion, we conducted a scenario analysis incorporating a PD-L1 testing cost of $224.09 USD per patient, which reflects the current average cost of PD-L1 testing in Chinese hospitals. The results showed that the ICER increased by only 2.37% from $9,458.80 to $9,682.91 USD per QALY in the overall population, which remains well below the WTP threshold. This confirms that excluding PD-L1 testing costs does not create a meaningful bias or alter our core conclusion.

The findings of this study provide robust evidence-based data to guide clinical decision-making, health insurance reimbursement policies, and the development of a tiered healthcare system for patients with HER2-negative advanced gastric cancer in China, offering substantial practical implications. In terms of clinical treatment selection, camrelizumab combined with CAPOX demonstrated marked cost-effectiveness advantages in both the overall population and the PD-L1-positive subgroup. Accordingly, camrelizumab combined with CAPOX is recommended as the preferred first-line treatment for patients with HER2-negative advanced gastric cancer in China. For healthcare institutions equipped to perform PD-L1 testing, this regimen should be prioritized for PD-L1-positive patients to maximize clinical benefit and economic efficiency; for institutions lacking PD-L1 testing capacity, direct use of this regimen remains cost-effective and clinically justified, thereby avoiding unnecessary treatment delays while awaiting test results. With respect to health insurance reimbursement, the results strongly support the inclusion of camrelizumab combined with CAPOX in the standard reimbursement list for first-line treatment of HER2-negative advanced gastric cancer. The substantial price reduction of camrelizumab achieved through national health insurance negotiations is a pivotal factor contributing to its cost-effectiveness. It is recommended that the current negotiated price level be maintained to ensure continued patient access. Additionally, differentiated reimbursement policies based on PD-L1 expression could be considered, offering higher reimbursement rates for PD-L1-positive patients to further optimize resource allocation within the health insurance system. Regarding the implementation of a tiered healthcare model, given the limited PD-L1 testing capacity in China’s primary care institutions, this study supports the adoption of the camrelizumab plus CAPOX regimen in primary care hospitals without mandatory PD-L1 testing. This approach can substantially increase the proportion of advanced gastric cancer patients receiving standardized immunotherapy at the primary care level and reduce the referral burden to tertiary hospitals. Tertiary hospitals should capitalize on their testing capabilities to implement PD-L1-based treatment stratification, thereby achieving an effective integration of precision medicine and cost containment.

Furthermore, the findings of this study provide valuable insights for future price negotiations of anticancer drugs. Drug pricing remains a critical determinant of the cost-effectiveness of immunotherapy. Further reductions in the prices of innovative drugs through national centralized procurement and health insurance negotiations represent the most effective strategy to enhance patient accessibility and alleviate the financial burden on medical insurance funds.

## 5 Conclusion

Camrelizumab combined with CAPOX chemotherapy for patients with HER2-negative advanced GC/GEJC delivers significant survival benefits and meaningful improvements in health-related outcomes, with an ICER well below the WTP threshold. Camrelizumab plus CAPOX therefore represents a cost-effective first-line therapeutic option for this patient population in the Chinese clinical setting.

## Data Availability

The original contributions presented in the study are included in the article/Supplementary Material, further inquiries can be directed to the corresponding author.
