# Supplementary material for: Camrelizumab plus CAPOX versus CAPOX for HER2-negative gastric or gastro-oesophageal junction adenocarcinoma: a cost-effectiveness analysis
Source: Front Pharmacol. 2026 Jul 8;17:1854496. doi: 10.3389/fphar.2026.1854496 (PMC13388140; doi:10.3389/fphar.2026.1854496)
Supplement: Supplementary file 1 [file DataSheet1.pdf]

Supplementary Table 1 CHEERS Checklist 2022.

Topic	No	Item	Reported
**Title**			
Title	1	Identify the study as an economic evaluation and specify the interventions being compared	Yes
**Abstract**			
Abstract	2	Provide a structured summary that highlights context, key methods, results, and alternative analyses	Yes
**Introduction**			
Background and objectives	3	Give the context for the study, the study question, and its practical relevance for decision making in policy or practice	Yes
**Methods**			
Health economic analysis plan	4	Indicate whether a health economic analysis plan was developed and where available	Not applicable
Study population	5	Describe characteristics of the study population (such as age range, demographics, socioeconomic, or clinical characteristics)	Yes
Setting and location	6	Provide relevant contextual information that may influence findings	Yes
Comparators	7	Describe the interventions or strategies being compared and why chosen	Yes
Perspective	8	State the perspective(s) adopted by the study and why chosen	Yes
Time horizon	9	State the time horizon for the study and why appropriate	Yes
Discount rate	10	Report the discount rate(s) and reason chosen	Yes
Selection of outcomes	11	Describe what outcomes were used as the measure(s) of benefit(s) and harm(s)	Yes
Measurement of outcomes	12	Describe how outcomes used to capture benefit(s) and harm(s) were measured	Yes
Valuation of outcomes	13	Describe the population and methods used to measure and value outcomes	Yes
Measurement and valuation of resources and costs	14	Describe how costs were valued	Yes
Currency, price date, and conversion	15	Report the dates of the estimated resource quantities and unit costs, plus the currency and year of conversion	Yes
Rationale and description of model	16	If modelling is used, describe in detail and why used. Report if the model is publicly available and where it can be accessed	Yes
Analytics and assumptions	17	Describe any methods for analysing or statistically transforming data, any extrapolation methods, and approaches for validating any model used	Yes

Characterising heterogeneity	18	Describe any methods used for estimating how the results of the study vary for subgroups	Not applicable
Characterising distributional effects	19	Describe how impacts are distributed across different individuals or adjustments made to reflect priority populations	Not applicable
Characterising uncertainty	20	Describe methods to characterise any sources of uncertainty in the analysis	Yes
Approach to engagement with patients and others affected by the study	21	Describe any approaches to engage patients or service recipients, the general public, communities, or stakeholders (such as clinicians or payers) in the design of the study	Not applicable
**Results**			
Study parameters	22	Report all analytic inputs (such as values, ranges, references) including uncertainty or distributional assumptions	Yes
Summary of main results	23	Report the mean values for the main categories of costs and outcomes of interest and summarise them in the most appropriate overall measure	Yes
Effect of uncertainty	24	Describe how uncertainty about analytic judgments, inputs, or projections affect findings. Report the effect of choice of discount rate and time horizon, if applicable	Yes
Effect of engagement with patients and others affected by the study	25	Report on any difference patient/service recipient, general public, community, or stakeholder involvement made to the approach or findings ofthe study	Not applicable
**Discussion**			
Study findings, limitations, generalisability, and current knowledge	26	Report key findings, limitations, ethical or equity considerations not captured, and how these could affect patients, policy, or practice	Yes
Other relevant information Source of funding	27	Describe how the study was funded and any role of the funder in the identification, design, conduct, and reporting of the analysis	Yes
Conflicts of interest	28	Report authors conflicts of interest according to journal or International Committee of Medical Journal Editors requirements	Yes

Supplementary Table 2. Comparison of survival models distribution（Overall population）

	AIC		BIC	
	Camrelizumab plus CAPOX group	CAPOX group	Camrelizumab plus CAPOX group	CAPOX group
OS				
Weibull	1056.675	2273.972	1063.027	2281.682
Log-normal	1041.427	2243.840	1047.779	2251.550
Log-logistic	1041.409	2242.844	1047.761	2250.555
Gompertz	1068.164	2299.094	1074.516	2306.805
Exponential	1068.220	2302.015	1071.396	2305.870
Gamma	1051.562	2262.191	1057.915	2269.902
PFS				
Weibull	956.206	1442.001	962.558	1449.711
Log-normal	926.484	1392.014	932.836	1399.724
Log-logistic	926.077	1388.248	932.429	1395.958
Gompertz	954.230	1475.043	960.582	1482.753
Exponential	955.906	1473.575	959.082	1477.430
Gamma	952.208	1421.457	958.560	1429.167

AIC: Akaike information criterion; BIC: Bayesian Information Criterion; OS: Overall survival; PFS: Progression-free survival;

Supplementary Table 3. Comparison of survival models distribution（PD-L1 positive population）

	AIC		BIC	
	Camrelizumab plus CAPOX group	CAPOX group	Camrelizumab plus CAPOX group	CAPOX group
OS				
Weibull	725.783	1515.979	731.472	1522.924
Log-normal	716.754	1497.100	722.442	1504.044
Log-logistic	714.543	1495.363	720.232	1502.308
Gompertz	730.917	1531.037	736.605	1537.981
Exponential	729.250	1532.095	732.094	1535.567
Gamma	723.161	1508.928	728.849	1515.872
PFS				
Weibull	643.572	968.929	649.260	975.873
Log-normal	625.191	929.802	630.879	936.746
Log-logistic	522.791	926.438	628.480	933.383
Gompertz	642.662	987.532	648.351	994.476
Exponential	642.804	985.532	645.649	989.004
Gamma	641.03	954.877	646.718	961.821

Supplementary Table 4. The base case results(weibull distribution)

Variable	Overall population		PD-L1 positive population	
	Camrelizumab plus CAPOX group	CAPOX group	Camrelizumab plus CAPOX group	CAPOX group
Cost (USD)	37465.20	17105.87	39538.39	17394.86
Incremental cost (USD)	20359.33	—	22143.53	—
QALYs	3.99	1.51	4.27	1.57
Incremental QALY	2.48	—	2.7	—
ICER (USD/QALY)	8209.41	—	8201.31	—

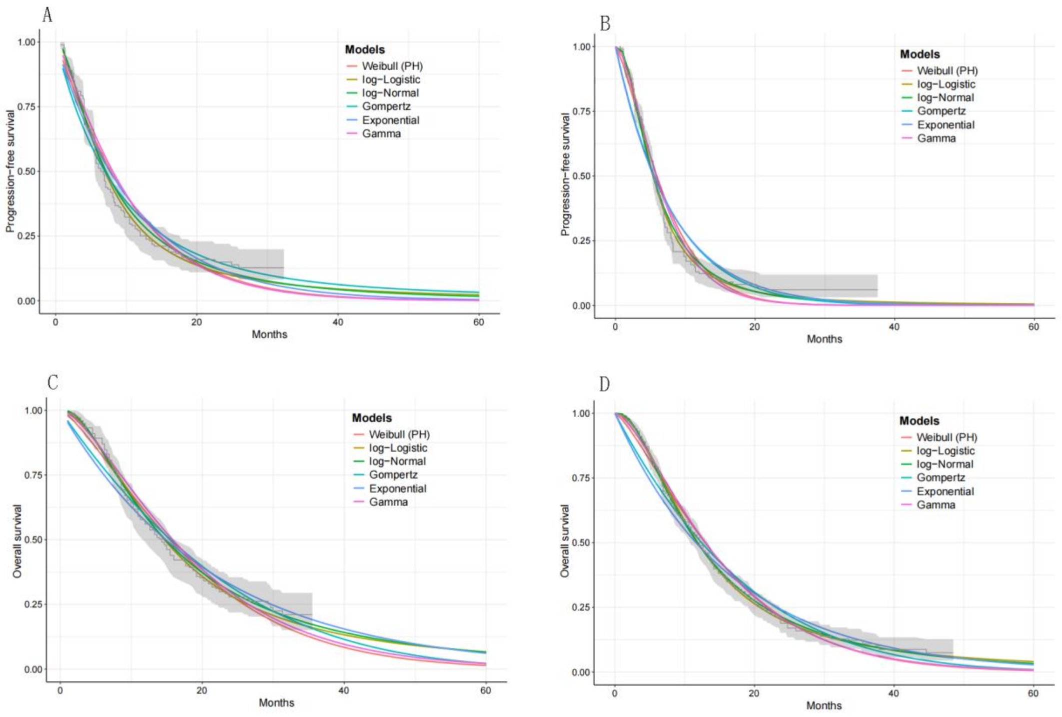

Supplementary Figure 1. The overall population survival curve distribution simulation A:Modes simulation visual progression-free survival curve of camrelizumab plus CAPOX group;B:Modes simulation visual progression-free survival curve of CAPOXgroup;C:Modes simulation visual overall survival curve of camrelizumab plus CAPOX group;D: Modes simulation visual overall survival curve of CAPOX group

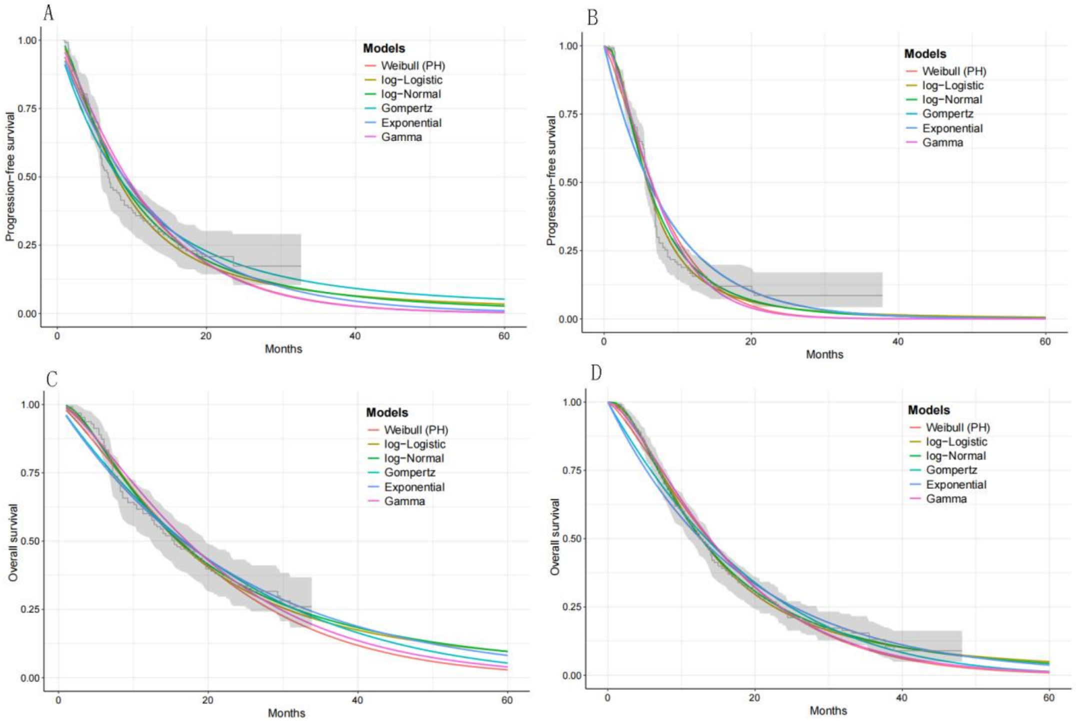

Supplementary Figure 2. The PD-L1 positive subgroup population survival curve distribution simulation

A:Modes simulation visual progression-free survival curve of camrelizumab plus CAPOX group;B:Modes simulation visual progression-free survival curve of CAPOXgroup;C:Modes simulation visual overall survival curve of camrelizumab plus CAPOX group;D: Modes simulation visual overall survival curve of CAPOX group

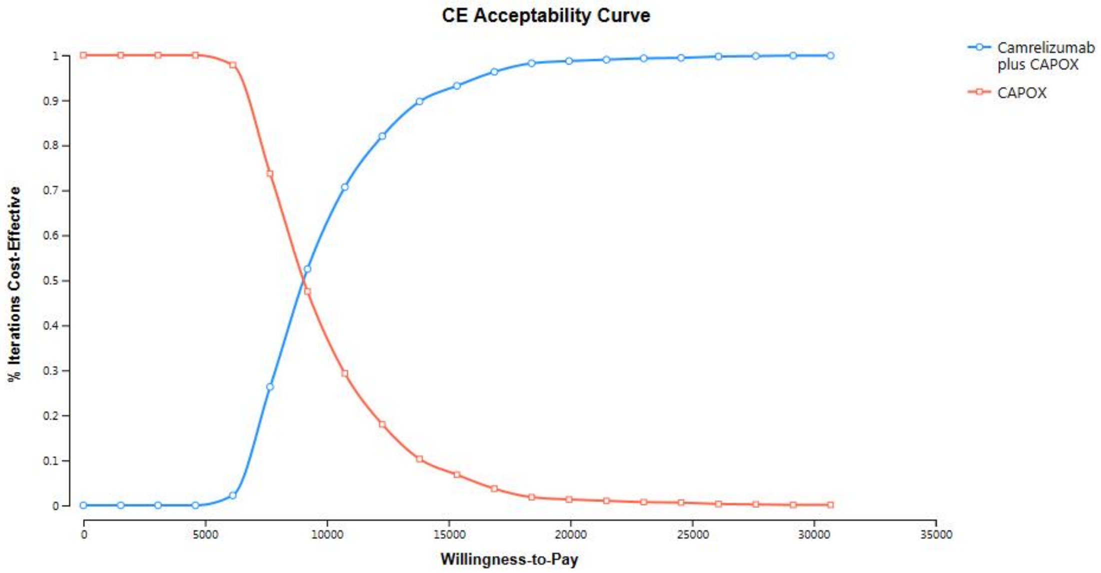

Supplementary Figure 3. The cost-effectiveness acceptability curve result for the PD-L1 positive subgroup

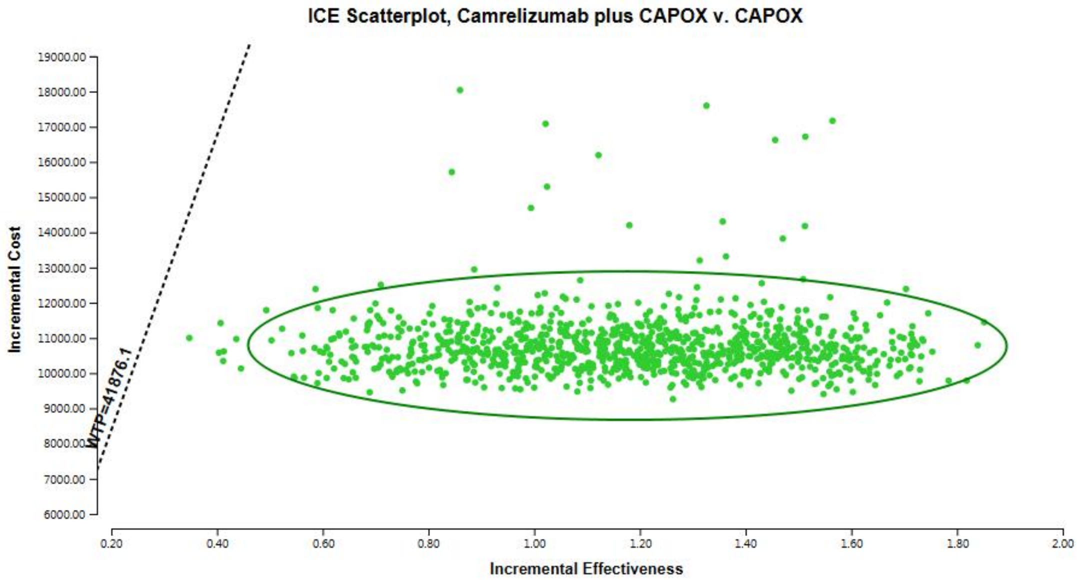

Supplementary Figure 4. The scatter plot result for the PD-L1 positive subgroup
